# Tooth-Supported Overdentures Revisited

**DOI:** 10.7759/cureus.53184

**Published:** 2024-01-29

**Authors:** Jia Zheng Leong, Yew Hin Beh, Ting Khee Ho

**Affiliations:** 1 Department of Oral and Maxillofacial Surgery, Hospital Tuanku Ja'afar, Seremban, MYS; 2 Department of Restorative Dentistry, Faculty of Dentistry, Universiti Kebangsaan Malaysia, Kuala Lumpur, MYS; 3 Division of Dentistry, School of Medical Sciences, Faculty of Biology, Medicine and Health, University of Manchester, Manchester, GBR

**Keywords:** geriatrics, complete dentures, removable dentures, precision attachment, tooth-supported overdenture

## Abstract

The goal of prosthodontics is to provide a functional prosthesis to restore aesthetics, functions, and masticatory efficiencies. Tooth-supported overdentures are one of the treatment options for removable dentures. This article aims to discuss the advantages and disadvantages of tooth-supported overdentures and the principles of using various overdenture attachments, including non-attachment overdentures. The common treatment options in preparing the overdenture abutment are either with or without abutment coping with or without attachment systems, which were discussed and illustrated. A range of tooth-supported overdenture systems were addressed, from low to high financial implications and treatment complexities. The clinician can choose a system that best fits the patient's condition and expectations. This allows clinicians to decide and consider tooth-supported overdentures as a treatment option before full edentulism. A well-executed tooth-supported overdenture ensures the preservation of alveolar bone, optimizes patient satisfaction in denture treatment, and eventually improves the patient's adaptation when transitioning to complete dentures.

## Introduction and background

Dental diseases are common among the elderly population and have resulted in tooth loss. With reduced functional dentition it greatly affects the patients, which causes them to experience reduced chewing ability, negative social impact, poor nutritional status, and mental health problems, thus affecting their overall quality of life [1]. The quality of life of this elderly cohort of the population can be improved by using a functional prosthesis tailored to individual needs [2]. Many prosthetic options are available for partially or fully edentulous patients, ranging from removable prostheses, fixed prostheses, implant-supported prostheses, and a combination of tooth implant-supported partial dentures. The outcome analysis comparing tooth-supported overdentures and implant-supported overdentures varied greatly.

Overdenture refers to removable dental prostheses that cover and rest on one or more remaining natural teeth, the roots of natural teeth, and/or dental implants [3]. Other nomenclature for this includes overlay dentures or overlay prostheses [3]. Tooth-supported overdentures are rather simple, avoid invasive surgical procedures, are more cost-effective, and there is evidence suggesting that tooth-supported overdentures wearers maintain better oral tactile sensibility due to preserved proprioception [4-7]. Due to these factors, this treatment option is preferred by many. On the other hand, implant-supported overdentures appeared to require less maintenance, offer better maximum bite forces, allow better chewing function, and exhibit higher abutment survival over time [8,9]. The biggest drawback of an implant-supported denture is the financial requirements. Having said that, each treatment option provided should take into account the patient’s medical status, anatomical considerations, and, more importantly, the financial standpoint, especially in a dependent elderly person [10].

There are various overdenture designs available, particularly tooth-supported overdentures, either with or without attachment systems, and each of them offers various advantages and disadvantages. A comprehensive overview of this topic with current knowledge is required for dental practitioners so this treatment can be adopted in their clinical practice. Therefore, this review aims to discuss the advantages and disadvantages of overdentures, particularly tooth-supported overdentures, and the principles of using various overdenture attachments, including non-attachment overdentures.

## Review

General clinical aspects of tooth-supported overdentures

Tooth-supported overdenture is indicated in patients with few remaining natural teeth that are periodontally healthy or with potentially reversible periodontal disease [11]. It is also recommended in patients with oral conditions such as xerostomia, a reduced height of the residual alveolar ridge, a high palatal vault, and unfavorable tongue positions or muscle attachments, which can negatively impact the stability and retention of the prosthesis [4]. When the remaining natural teeth are not in a favorable position or morphologically compromised to support a fixed partial denture (FPD) or removable partial denture (RPD), the teeth can be modified to be used as abutments to support an overdenture [12]. In rare situations, such as oligodontia [13], cleft palate, or tooth developmental anomalies that affect the coronal structure, such as dentinogenesis imperfecta or amelogenesis imperfecta, they may benefit from receiving an overdenture [5].

In patients who received a significant dose of head and neck radiation or received bisphosphonate or anti-angiogenic medication, maintaining a devitalized natural tooth root is considerably beneficial to avoid the potentially debilitating effects of osteoradionecrosis or medication-related osteonecrosis, respectively [14,15]. Although the reported incidence of developing osteoradionecrosis after tooth extraction was only about 7%, the management of the condition is unpredictable [16].

Despite the benefits of a tooth-supported overdenture, some of the factors may contra-indicate such treatment. From a patient’s perspective, poor motivation to maintain good oral hygiene, poor systemic health, financial restriction, level of dependency, and accessibility to treatment facilities hinder the delivery of such treatment [17]. The local factors include the insufficient inter-arch space that is required for setting up the denture teeth, especially at the location where additional space is required to fit the abutment restorations or precision attachment systems. If the space is limited, it could potentially result in thin acrylic denture bases and a risk of denture fracture [11].

Several advantages and disadvantages of tooth-supported overdentures were widely described in the literature [4-7,11,12,18-21] and summarized in Table 1. To ensure a successful overdenture prosthesis, a careful selection of an abutment tooth is essential. The criteria for abutment selections are summarized in Table 2 [22-24].

**Table 1:** Advantages and disadvantages of tooth-supported overdentures

Advantages	Disadvantages
Maintenance the alveolar bone height thereby increases the retention, stability, and support of the denture.	Adjunctive treatments are required, such as endodontic treatment, abutment restoration, root caps, or precision attachment.
Preservation of proprioception within the periodontal ligament, which helps in regulating the biting force over the denture.	Additional number of treatment visits.
Reduction of the coronal portion of the abutment with a reduced but healthy periodontal support will improve the crown-to-root ratio and prolong the survival of the abutments.	It is expensive to provide endodontic treatment and fabricate cast copings or precision attachments.
Transitional situation if the patient becomes fully edentulism later on.	Prominent bony undercuts in the area of abutment teeth, especially in the mandibular anterior, may prevent full extension of the denture’s flange and eventually compromise the retention and aesthetic outcome.
	Risk of having a thin acrylic denture base, which eventually leads to denture base fracture.
	The challenges for tooth set-up are due to the space required for abutment attachment systems, especially in cases with limited inter-occlusal space.
	Scheduled recall appointments are needed to reinforce patients' oral hygiene measures and the application of topical fluoride to prevent caries and periodontal disease.

**Table 2:** The criteria for a predictable abutment selection

Consideration factors	Criteria
Periodontic consideration	Periodontally healthy or a tooth and a reduced but healthy periodontium and a support of at least 50% of alveolar bone. The abutment mobility should be minimal.
Endodontic considerations	Preferably a single-rooted tooth with root canal morphology that eases root canal treatment.
Structural considerations	The abutment should be restorable, having sufficient supragingival tooth structure.
Abutment location/distribution considerations	The abutment should have at least one tooth in each quadrant (commonly canine or premolars), and the roots should be ideally symmetrically distributed.

Preparation of abutment teeth for tooth-supported overdenture

A tooth-supported overdenture can be incorporated into a removable partial denture or a complete denture. The tooth abutments require preparation before they are fully functional in supporting a prosthesis. There are several options for preparing the abutments [11,25]. Some of these options are as follows:

Simple Tooth Modification or Non-Coping Overdentures

**Figure 1:**
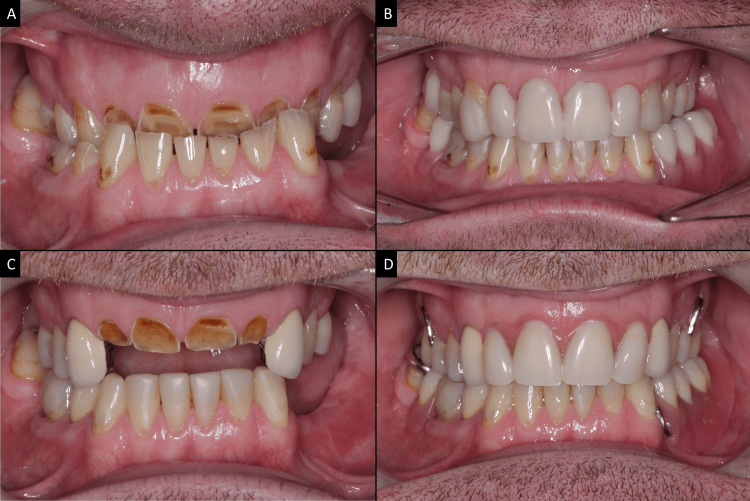
Simple tooth modification or non-coping overdentures (A) The patient presented with partial edentulism and severe pathological tooth surface loss with occlusal curve disharmony. (B) Provisionalization phase with direct composite build-ups and acrylic overdentures using teeth 12 to 22 as abutments at an increased occlusal vertical dimension (OVD). (C) Vital overdenture abutments 12 to 22. Note that any sharp coronal structure of the maxillary incisors was selectively ground and polished. (D) Cobalt-chromium-based maxillary removable partial overdenture and mandibular removable partial denture (RPD). Image credits: Ting Khee Ho

This type of tooth modification is often used in patients with advanced physiological or pathological tooth wear where the teeth are vital but have fewer pulpal responses due to the reduction in dentinal tubule diameter and pulpal recession [22,26]. These teeth have a significant amount of tertiary dentin to protect the pulp despite the severe coronal tooth surface loss. The teeth can be reshaped to eliminate the undercut and alter the coronal height without encroaching on the pulpal chamber, allowing space for the denture tooth placement (Figure 1). The occlusal surface may be restored with glass ionomer cement or resin composite to protect the abutments or simply left without restoration [27]. This is the simplest, with the least cost and time-consuming procedure.

 

Endodontic Treatment With Direct Restoration

**Figure 2:**
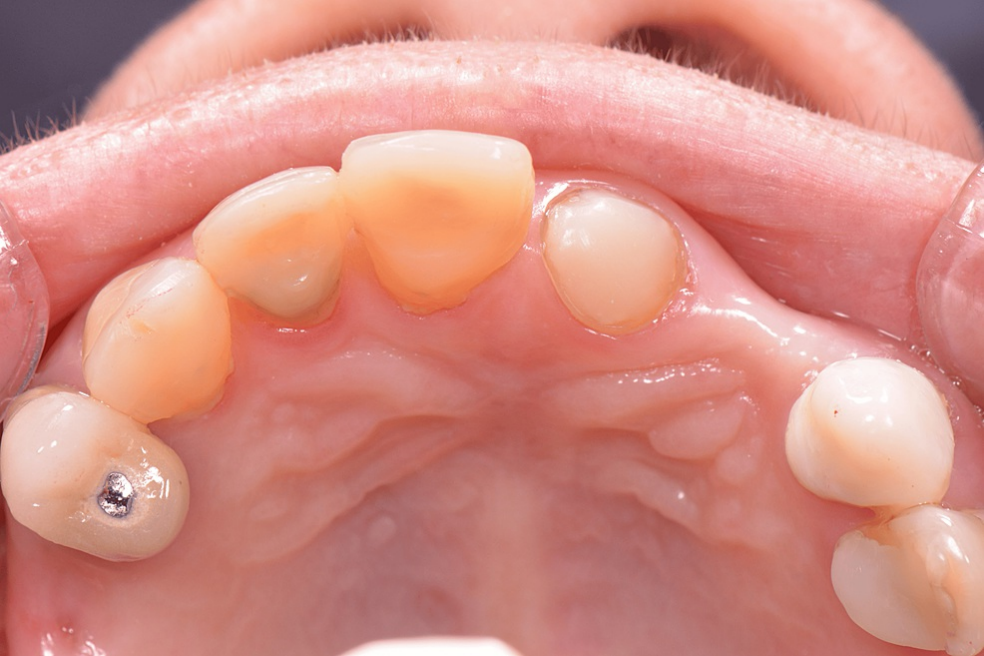
Abutment with endodontic treatment and direct composite restoration Tooth 21 was endodontically treated, and a direct composite restoration was used as a coronal seal to support a removable partial overdenture. The restoration was made in a dome shape, eliminating any undercut around the abutment. Image credits: Yew Hin Beh

In this approach, the tooth will undergo an elective endodontic treatment, followed by a reduction of the crown height to near the gingival level. The root canal orifices are restored with an amalgam plug, glass ionomer cement (GIC), or composite resin restoration (Figure 2). Keltjens et al. [28] reported that the survival rate of an amalgam, resin modified GIC, and resin composite overdenture abutment restoration was not statistically different after four years of observation. It is advisable to restore the root canal orifice under dental dam isolation before the reduction of crown height to the gingival level, which may preclude abutment isolation issues and increase the success rate of the restoration and the endodontic treatment.

Endodontic Treatment With Cast Coping

**Figure 3:**
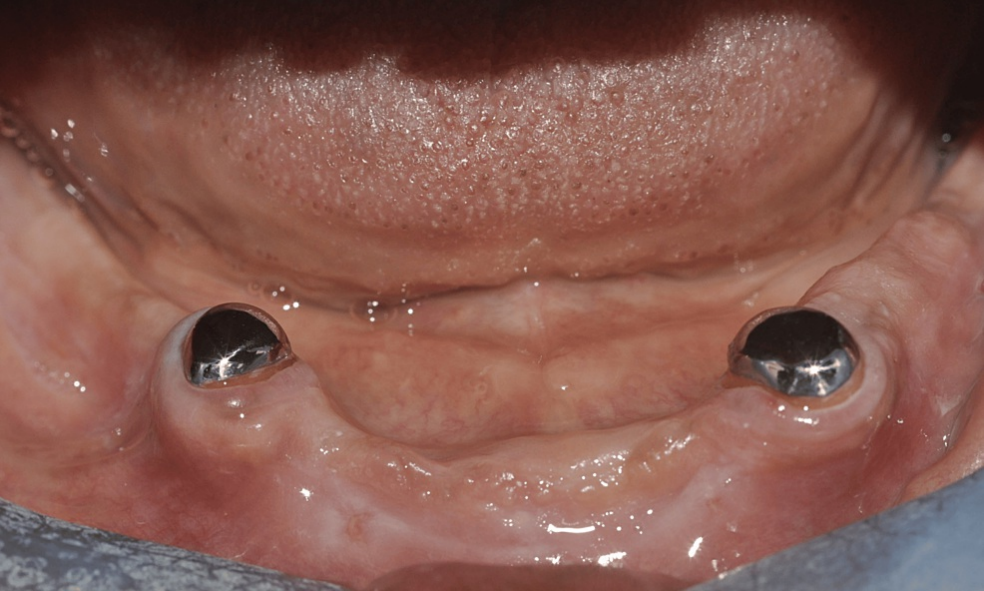
Cast coping overdentures Tooths 33 and 43 were endodontically treated and prepared to receive a metal cast to cope with intra-radicular retention. Image credits: Ting Khee Ho

A cast coping can be made using a base metal, a semi-precious alloy, or a precious alloy. The root-treated abutment will be decoronated, leaving a 2mm ferrule height, and prepared circumferentially to receive cast metal coping [7]. The cast coping can be extended into the root canal with or without a post, depending on the amount of tooth structure available. Post-retained coping may be extended into the root canal for at least 2/3 of its root length to enhance retention of the coping (Figure 3) [6]. These copings are used to protect a weakened tooth from fracture and excessive wear. Current data has proven that this type of abutment has excellent survival, with a reported 1.76% risk of abutment loss annually [29]. Nevertheless, the idea that coping reduces the incidence of secondary caries on the abutment remains controversial, and caries and periodontal disease were reported as the most common etiologies for abutment loss [24,29].

Double Crown-Retained Overdentures

The double crown-retained overdentures refer to a type of removable prosthesis that utilizes two units of crowns as retaining elements and can be either in telescopic or conical form [30]. These retainers consist of a primary crown cemented onto the abutment tooth and a secondary crown that is incorporated onto the overdenture [30]. The primary crown has a convergence ranging from parallel up to 8°, allowing a wedge action and friction hold between the primary and secondary crowns, which eventually exerts a retaining force of around 5-14N [30,31]. With this telescopic double crown abutment system, the overdenture can be designed as an open flange, allowing enhanced cleanability and improving salivary flow, leading to reduced plaque retention. This feature considerably reduced its biological complications on the abutment tooth [6]. This type of overdenture avoids unsightly clasp-retained removable dentures, particularly for an aesthetically concerned patient [6]. On the other hand, the space requirements were rather meticulous to accommodate two crowns in a single abutment, rendering more tooth preparation and potentially requiring endodontic treatment prior [32].

In a recent study, 14% of the double crown’s abutment was reported to have fractured during the observation period, and only a small number of them are salvageable [33]. However, another report showed no abutment loss in 84% of the study population after five years [32]. Despite the enhanced denture retention, the retentive forces reduced after long term use due to the loss of friction hold between the primary and secondary crowns. This was less evident in a double crown with additional retentive features [31]. Furthermore, abutment with a resilient double crown showed slightly higher survival; however, this was not statistically significant [32].

Attachment Retained Overdentures

A precision attachment is defined as a retainer consisting of a metal receptacle (matrix) and a closely fitting part (patrix). The matrix is contained within the expanded contours of the abutment crown or dental implant, and the patrix is attached to a pontic or a removable partial denture (RPD). It can also be defined as an interlocking device to connect one component, which is attached to the abutment, and another end into an RPD with the role of retaining or stabilizing it [3]. The male part (matrix) can be soldered to the abutment coping or directly cemented onto the abutment root canal [34,35]. The matrix, depending on the material and system, consists of a nylon cap with various predetermined retention forces that can be individually selected based on the needs of the patient. This particular nylon cap is subjected to wear and tear and requires replacement when it has lost its retention force [35,36]. An in-vitro study shows a significant loss of retention of the nylon cap after two years in service [36].

The precision attachment of retained overdentures has shown many advantages over conventional removable overdentures. It has significantly improved the retention, support, and stability of the denture, facilitating the management of divergent abutments and potentially improving the aesthetic by avoiding visible retainers in the aesthetic zone [6,34,37]. The survival rate of such abutment was high and is reported to be 95% after five years and 88% after 10 years [6]. However, the disadvantages are the cost associated with the increased laboratory stages and the required technician skills, the necessity to prepare the abutment teeth, a minimum of 4 mm of vertical inter-occlusal space required, and a huge effort from both clinician and patient for maintenance [34,38]. The most common complications of precision attachment retained overdentures were still caries and periodontal disease [6].

Generally, precision attachments in overdentures are radicular attachments. The radicular attachments that are commonly used in overdentures are studs, bar attachments, and magnetic attachments [6,34,37].

**Figure 4:**
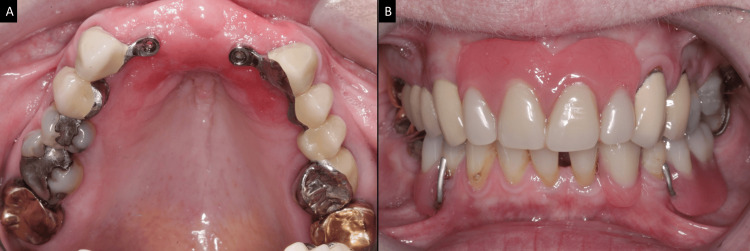
CEKA attachments (A) CEKA attachments cast on porcelain fused to metal-splinted crowns on the maxillary teeth to provide retention for the RPD. CEKA attachments use a spring pin that snaps accurately into the female components, providing direct retention and eliminating an unsightly direct retention clasp on the aesthetic region. (B) Clinical photo of a cobalt-chromium maxillary removable partial overdenture in situ. Image credits: Ting Khee Ho

**Figure 5:**
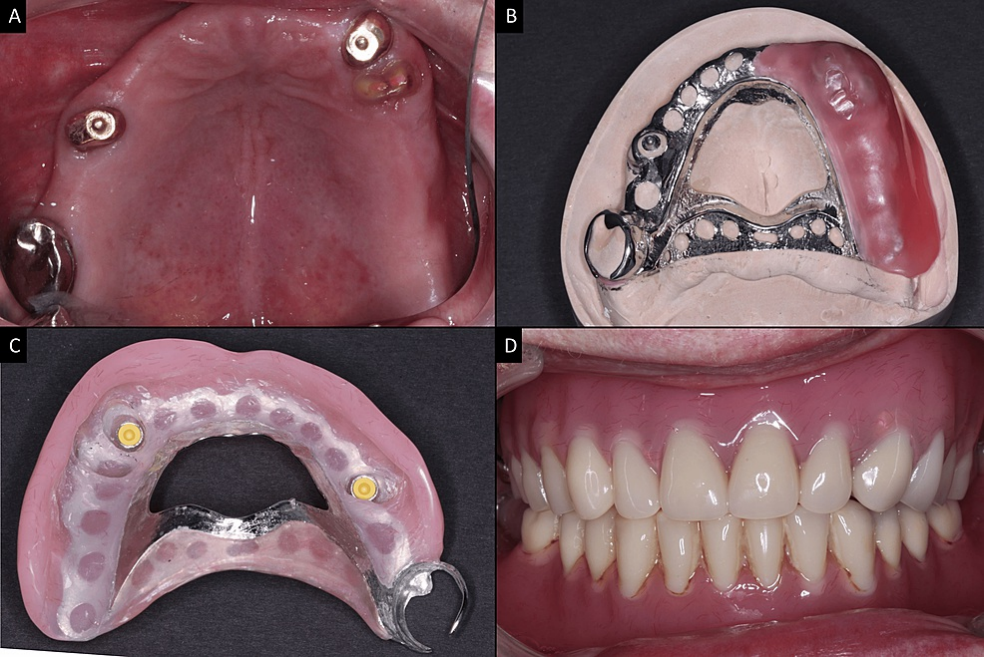
Rhein OT equator castable attachment system (A) Stud overdenture attachment systems (Rhein) on abutment teeth 15 and 23 are cast with gold alloy coping. (B) Cobalt-chromium framework. (C) Completed overdenture with retentive nylon attached to the denture base. (D) Clinical photo of cobalt-chromium upper removable partial overdenture in situ. Image credits: Ting Khee Ho

The two main types of studs are the intra-radicular and extra-radicular studs. For the intra-radicular stud, the housing or female part is located within the root surface, and the stud (male element) is fabricated on the denture base. It is recommended in clinical situations with reduced inter-occlusal height [5]. Extra-radicular studs are more popular among dental practitioners. The stud (male element) is usually attached to the metal coping cemented onto the endodontically-treated root, and it projects from the root surface, while the housing (female element) is incorporated into the denture base. These studs are available in different forms, such as prefabricated attachments or plastic patterns. The prefabricated attachment can be soldered directly to the cast metal coping, whereas the plastic patterns are attached to the waxed-up copings on the abutments that were to be casted together. The use of a prefabricated attachment is limited by its fixed angulation. Surveying the plastic patterns on waxed-up copings can ensure the parallelism of the studs for a single path of insertion. Examples of attachment systems are the CEKA (Figure 4A), Rhein OT Equator Castable (Figure 5A), O-Ring, Dalbos, Rotherman, and Gerber attachments. Stud systems like the Rhein OT Equator Attachment consist of a sphere with a flat head available in preformed plastic patterns that are cast to copings on abutments and female housing (nylon rubbers) (Figure 5C) in different colors corresponding to different retention degrees ranging from 0.6 kg to 2.5 kg retention force. A retrospective study on the survival of the abutment teeth with precision attachment demonstrated that 86.2% of the capped abutment teeth were still intact after 14 years [6].

Bar attachments provide retention, horizontal stability, and support for an overdenture by splinting two or more abutments together. A common bar attachment construction consists of one piece of cast bar linking two cast copings that are cemented onto the endodontically-treated roots, which are normally the canines [34]. A clip or sleeve is held in the denture, which provides retention for the denture. Bar attachments can be used with divergent root canals to overcome non-parallel abutments. One of the most important steps in choosing a bar attachment is to assess the space adequacy to house the component in the denture by considering the vertical relationship, occlusion, and contour of the prostheses. The space requirements are dependent on the bar attachment system utilized, and each system has its own space requirements [34]. Despite its effectiveness, it requires meticulous oral hygiene care, as these bars can complicate effective plaque removal under the bar system, leading to abutment loss [5]. Some common examples of bar overdenture systems are the Hader bar, Dolder bar, Ackermann bar, and CM bar.

**Figure 6:**
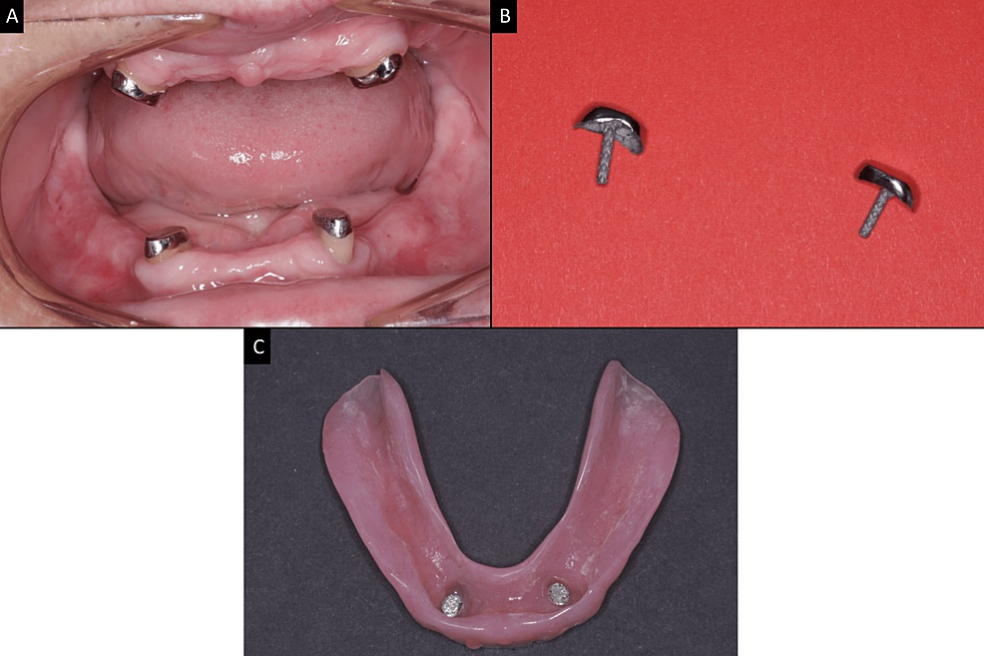
Cast coping magnetic attachment system (A) Casted magnetic coping cemented on abutments. (B) Casted magnetic coping with intra-radicular retention prior to cementation. (C) The magnetic attachment on the intaglio surface of the mandibular complete denture.  Image credits: Ting Khee Ho

The magnetic attachment system consists of a magnetic keeper and a prosthetic retention element [37]. The keepers are the root cap components, which can be broadly classified into cast coping or resin coping types. For the cast coping type, the keeper is incorporated into the cast metal post and later cemented to the endodontically treated abutment (Figure 6).

**Figure 7:**
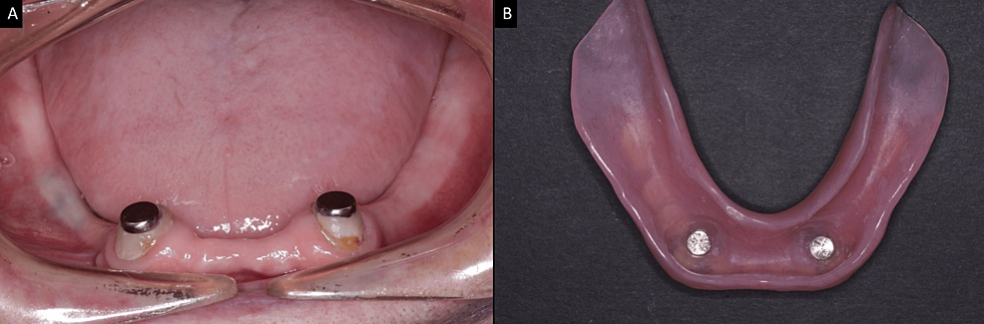
Prefabricated magnetic attachment system (A) Magnetic component directly cemented on the endodontically-treated abutments. (B) The retention units are located on the intaglio surface of the mandibular complete denture. Image credits: Ting Khee Ho

The resin coping type utilizes a direct adhesion technique where the keeper is cemented to the root by resin cement and secured with resin composite around it (Figure 7). The prosthetic retention element consists of rare earth Samarium-cobalt (SmCo) magnets or Neodym-iron-boron (NdFeB) magnets [39]. This magnetic attachment is relatively less demanding on dental laboratory support compared to other precision attachments. It is also suitable for elderly patients who have reduced manual dexterity, as the denture will snap in when wearing it and snap out when removing it [37]. Although the retention force is comparable to other non-magnetic attachment overdenture systems, the magnetic force is retained longer [37]. Hence, less maintenance and replacement of components. Other advantages include strong retention forces owing to the small size of the magnetic piece, automatic reseating of the denture to its attachment, less demand on the parallelism of the abutments, ease of home care, and also the fact that the alveolar bone support on the abutment tooth is less critical [37]. On the other hand, clinically, magnetic retention diminishes over time. However, the main causative reason for this was not the diminishing magnetic force, which is rather constant over time, but the increasing gap between two magnetic pieces over time [34,37,39]. The metal corrosion from the metal pieces was rather concerning in the long run [5]. 

Complications of overdentures

The goal of prosthodontics is to provide a functional prosthesis, which could improve oral well-being. For mandibular complete denture wearers, adapting to the denture can be difficult owing to the natural anatomy of the mandible, which often fails to provide adequate denture support, retention, and stability. The basis of overdenture is the ability to preserve the residual alveolar ridge height, thereby optimizing the retention and support of the denture [40]. A good attachment-prosthesis relationship and a proper selection of attachments will enhance the patient’s adaptability to the denture. Nevertheless, adherence to the basic principles of complete denture design is paramount to ensuring the success of the denture. This includes a proper border seal that is within the limitations of functional muscle movement, extension of the denture to the retromolar pad, an accurate maxilla-mandibular record, and occlusion [41].

Complications of overdenture can be classified into two categories: biological and technical complications. A systematic review reported that the most common biological reasons for loss of abutment teeth with cast copings are caries and periodontal disease, with prevalence ranging from 0.5% to 83% and from 4% to 86%, respectively, whereas the prevalence of tooth mobility and tooth fracture ranges from 1.0% to 1.7% [29]. Ettinger and Qian [22] reported an over-denture abutment loss of up to 20% in their prospective study with up to 22 years of follow-up. The role of topical fluoride in controlling carious lesions has been inconclusive. However, efforts by the patients to maintain good cleaning procedures and maintenance procedures every six months by the clinician are of utmost importance [22,42]. Even so, other considerations when treating the older cohort of patients would be manual dexterity and also eyesight, which reduce significantly as they age, not to mention patients with musculoskeletal disorders or neurological disorders that prevent good self-oral hygiene care [10]. In conditions where the caries’ risks are high, an implant-supported or implant-retained prosthesis is much more predictable [43]. The other biological compilations include denture stomatitis, fracture of abutment teeth, and apical lesions on abutment teeth [35].

In terms of technical complications, an early complication could happen from a less experienced dental laboratory technician. When there is an excessive space in the prosthesis around the gingival margin, a “dead space” may develop and lead to gingival hypertrophy. However, a displacement wash in the final impression could minimize such problems [41]. An unsatisfactory attachment-prosthesis relationship renders denture relining difficult and time-consuming. This is especially true in bar attachment due to the presence of an undercut below the bar [44]. Hence, reconstruction of a new denture is recommended.

Over time, alveolar bone resorption or occlusal wear of the abutment or attachments (most commonly the matrix portion of the stud abutment) will take place, resulting in the improper fitting of the denture with the abutment tooth and residual alveolar ridge [45]. Such situations allow the denture to rock around the abutment teeth, causing discomfort, occlusal and denture retention problems, as well as denture base fractures. Retention loss of the matrix is most common in mandibular overdentures. When retention is unrelated to the matrix, but there is a problem with the precision attachment of the root cap, the construction of new sets of dentures or the replacement of the root stump with an implant is recommended [35]. Moreover, to reduce the rate of denture base fracture, metal framework reinforcement is recommended, especially in abutments with precision attachment.

Other mechanical complications are decementation of cast coping, coping remakes, denture base fracture, chipping of cast coping or attachment, and post-fracture [29]. Root fractures were commonly observed in overdentures that were supported by less than three abutment teeth as a direct consequence of overloading. On the other hand, when an overdenture was supported by more than three abutments, denture base fractures were more often observed. This is due to the reduced denture base thickness around abutment teeth because of the space limitation [35].

There are a variety of attachments on the market, but it is crucial to select the most appropriate attachment for a specific clinical situation. The knowledge of the distribution of the occlusal force transmission to the residual ridge and the abutment with attachments ensures higher clinical success. The principles in selecting attachments for overdentures include the availability of supporting bone, the patient’s prosthetic expectation, the patient’s financial ability, the clinician’s choice and clinical expertise, and the laboratory technician’s skills and experiences. Nevertheless, one should also consider the regular maintenance cost of any replaceable parts that were subjected to wear and tear over years of usage [35].

## Conclusions

In summary, tooth-supported overdenture is the last resort before rendering the patient to full edentulism. Considering the benefits of overdentures, instead of extracting the terminal dentition, careful consideration shall be given to the potential role of the tooth or teeth as an overdenture abutment. With the wide array of different types of tooth-supported overdenture systems discussed, from the low to high financial implications and treatment complexities, clinicians can choose a system that best fits the patient's condition and expectations. This allows a smooth transition to complete dentures, improving patients’ adaptability, which, in the long run, significantly benefits the patient. A carefully planned tooth-supported overdenture leads to increased patient satisfaction and improved oral health-related quality of life. 
